# Investigation of Etiologic Agents and Clinical Presentations of Otomycosis at a Tertiary Referral Center in Tehran, Iran

**Published:** 2019-02

**Authors:** Hasti KAMALI SARWESTANI, Roshanak DAIE GHAZVINI, Seyed Jamal HASHEMI, Sassan REZAIE, Mohsen GERAMI SHOAR, Shahram MAHMOUDI, Miad ELAHI, Ardavan TAJDINI

**Affiliations:** 1. Department of Medical Parasitology and Mycology, School of Public Health, Tehran University of Medical Sciences, Tehran, Iran; 2. Students’ Scientific Research Center, Tehran University of Medical Sciences, Tehran, Iran; 3. Department of Medical Parasitology and Mycology, Faculty of Medicine, Urmia University of Medical Sciences, Urmia, Iran; 4. Department of Head and Neck Surgery, Amir Alam Hospital, Tehran University of Medical Sciences, Tehran, Iran

**Keywords:** Otitis externa, *Aspergillus niger*, *Candida glabrata*, Iran

## Abstract

**Background:**

Otomycosis is a superficial infection of the ear caused by a spectrum of various fungal agents and its epidemiology depends on geographical region and climatic condition. The aim of this study was to investigate the causal agents and clinical manifestations of otomycosis at a tertiary referral center in Tehran, Iran.

**Methods:**

From Apr 2016 to Jan 2017 a set of 412 subjects with suspicion of external otitis were included. Clinical examination and specimen collection were performed by an otorhinolaryngologist. Subsequently, direct examination and culture were performed on specimens and isolated molds were identified morphologically. Yeast isolates were identified using CHROMagar *Candida* medium and PCR-RFLP of ribosomal DNA whenever needed. Data were analyzed using SPSS.

**Results:**

Otomycosis was confirmed in 117 cases (28.39%) including 64 (54.7%) males and 53 (45.3%) females. Patients were within the age range of 10–75 yr and the highest prevalence was found in the age group of 46–55 yr (30.77%). Pruritus (89.74%) and auditory manipulation and trauma (83.76%) were the predominant symptom and predisposing factor, respectively. Among 133 isolates from 117 patients, *Aspergillus niger* (n=50, 37.59%) was the most common etiologic agent and *Candida glabrata* (n=25, 18.8%) was the predominantly isolated yeast. Furthermore, 16 cases of mixed infection were identified and coinfection due to *A. niger* and *C. glabrata* (seven cases) was the predominant pattern.

**Conclusion:**

Our results revealed the high prevalence of *C. glabrata* and mixed infections in otomycosis patients. Therefore, mycological examinations should be considered for proper treatment.

## Introduction

Otomycosis is a superficial fungal infection of the ear involving the external auditory canal, auricle and even tympanic membrane (1, 2). The causative agents include a wide spectrum of saprophytes (hyaline and dematiaceous), yeasts and rarely dermatophytes (3–5). Although more than 50 species of fungi, including *Penicillium* spp., *Fusarium* spp., Mucoracae, *Scopulariopsis* spp., *Alternaria* spp., *Malassezia* spp., *Candida* spp. and dermatophytes have been reported as pathogenic agents of otomycosis (6–8). However, most studies reported *Aspergillus niger* and *Candida albicans* as the most common causes of otomycosis (7, 9).

The prevalence of otomycosis among all cases of otitis externa is reported to be more than 10% (8). Although both genders and all age groups are affected by otomycosis, this condition is more common in the Middle Ages (10, 11).

Otalgia, itching, hearing loss, ear fullness, and tinnitus are the most common clinical manifestations of fungal otitis (12–14). The prevalence of otomycosis and its fungal agents varies in different geographical areas (3) and can be influenced by several predisposing factors such as hot and humid climate, swimming, traumatic injuries or manipulation of the ear canal and also bacterial infections (15, 16).

Azoles, such as clotrimazole, fluconazole, and miconazole are more effective than other anti-fungal drugs in treatment of otomycosis and apparently, they have no ototoxic properties (17). Meanwhile, proper aural hygiene and cleaning the discharges and debris in the ear seems to be effective in topical treatments (17). Furthermore, regarding the recurrence of disease due to insufficient and inadequate treatment, proper identification of the pathogenic agents could be helpful in finding the accurate therapeutic strategies (17). Accordingly, the present study carried out to determine the prevalence of otomycosis and its etiological agents in patients with suspected otitis externa, and to determine the predisposing factors and clinical manifestations of patients in a tertiary referral center in Tehran, Iran.

## Material and Methods

### Patients

This descriptive cross-sectional study was conducted from Apr 2016 to Jan 2017 on patients referred to the AmirAlam Hospital as a tertiary referral center affiliated to Tehran University of Medical Sciences, Tehran, Iran. All subjects with clinical symptoms indicative of otitis such as otalgia, itching, discharge met the inclusion criteria for enrollment in this study.

All patients read, understood and signed the informed consent statement and the present study was approved by the Ethics Committee of Tehran University of Medical Sciences (Ethics code: IR.TUMS.SPH.REC.1395.1793).

Demographic data, clinical history and manifestations, and underlying factors of all individuals were recorded through questionnaires and clinical examinations. Subsequently, specimen collection was performed by an otolaryngologist using sterile swabs. The specimens immediately were transferred to the Medical Mycology Laboratory, School of Public Health, Tehran University of Medical Sciences, Tehran, Iran, for further investigations.

### Direct examination and culture

Direct microscopic examination was performed on all specimens using 10% KOH. Furthermore, all samples were cultured on the sabouraud dextrose agar (SDA, Merck, Germany) and SDA supplemented with chloramphenicol (QUELAB, UK) and incubated at 30 °C for 20 d. All culture plates were checked daily in the first week and afterward at least three times a week. Mold isolates were identified based on the microscopic and macroscopic characteristics of colony. In order to identify *Candida* species, CHROMagar Candida medium (CHROMagar, France) was utilized.

### Molecular identification

*Candida* isolates with ambiguous colony colors on CHROMagar Candida medium were identified using PCR-RFLP of the internal transcribed spacer regions of rDNA. In this regard, primers ITS1 (5′-TCC GTA GGT GAA CCT GCG G-3′) and ITS4 (5′ –TCC TCC GCT TAT TGA TAT GC-3′) and restriction enzyme *MspI* were used (18).

### Statistical analysis

Data were analyzed using SPSS software ver. 15 (Chicago, IL, USA). In order to determine the association between different predisposing factors with the occurrence of otomycosis, Chi-square test was performed. *P*<0.05 was considered statistically significant.

## Results

**Fig. 1:**
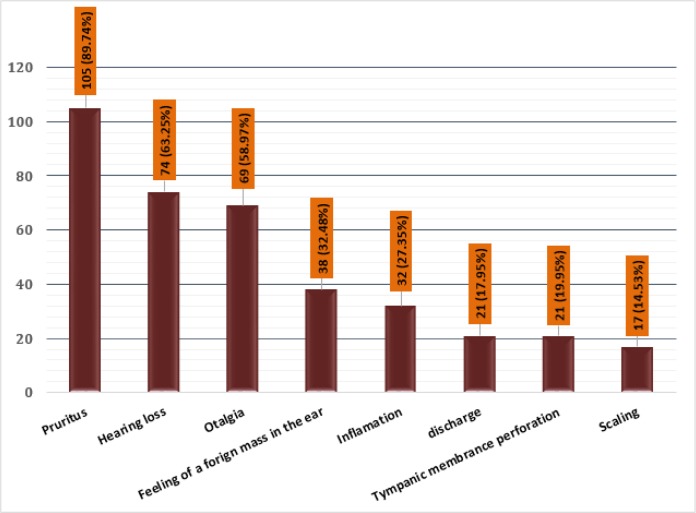
The frequency of different clinical signs and symptoms among 117 patients with otomycosis

Totally 412 patients with a suspicion of otitis externa in the age range of 9 to 75 yr were enrolled. Of them, 117 (28.39%) patients including 64 (54.7%) males and 53 (45.3%) females were confirmed for otomycosis. The patients were within the age range of 10–75 yr and the highest prevalence of otomycosis was found in the age group of 46–55 yr. Table 1 represents the distribution of patients based on their gender among different age groups. Among these cases, pruritus was the most common symptom (89.74%) and scaling had the lowest prevalence (14.53%) (Fig. 1).

**Table 1:** The distribution of 117 patients with otomycosis based on their gender in different age groups

***Patients****(N=117)*	***Gender***	***Age ranges (yr)***								
		***≤15***	***16–25***	***26–35***	***36–45***	***46–55***	***56–65***	***66–75***
Positive cases (n) (%)	Male	1 (0.85)	6 (5.13)	15 (12.82)	11 (9.4)	19 (16.24)	10 (8.55)	2 (1.71)
	Female	0	3 (2.56)	12 (10.25)	10 (8.55)	17 (14.53)	7 (5.98)	4 (3.41)
Total (n) (%)	-	1 (0.85)	9 (7.69)	27 (23.07)	21 (17.95)	36 (30.77)	17 (14.53)	6 (5.12)

Trauma/manipulation of the ear canal, history of bacterial infection and swimming were found in 83.76%, 33.33%, and 30.77% of patients, respectively. These predisposing factors were significantly associated with the occurrence of otomycosis (*P*<0.001).

Based on the results of culture and molecular analysis (PCR-RFLP), *A. niger* (37.59%) was the dominant etiology of otomycosis in our study. Moreover, *C. glabrata* was the most frequent *Candida* species (18.8%). The spectrum of fungal species and their frequency are presented in Table 2. Furthermore, mixed fungal otitis was observed in 16 of 117 patients (13.67%). The majority of mixed cases were due to *A. niger* and *C. glabrata* (7 patients) followed by *A. niger* and *A. flavus* (4 patients) (Table 3).

**Table 2:** The spectrum of isolated fungi from 117 patients with otomycosis

***Fungi***	***Frequency***	***Percent***
*Aspergillus niger*	50	37.59
*Aspergillus flavus*	36	27.06
*Candida glabrata*	25	18.79
*Candida albicans*	7	5.26
*Candida tropicalis*	5	3.76
*Penicillium* spp.	4	3
*Candida parapsilosis*	2	1.5
*Malassezia* spp.	2	1.5
*Mucor* spp.	1	0.75
*Rhizopus* spp.	1	0.75
Total	133^a^	100

a. Mixed infections were observed in 16 patients.

**Table 3:** The different patterns of mixed fungal otitis due to *Aspergillus* and *Candida* species observed among 117 patients with otomycosis

***Fungal agents***	***Number of patients***
*A. niger* + *C. glabrata*	7
*A. niger* + *A. flavus*	4
*A. flavus* + *C. glabrata*	2
*A. niger* + *C. parapsilosis*	1
*A. flavus* + *C. albicans*	1
*A. flavus* + *C. tropicalis*	1
Total	16

## Discussion

We evaluated the prevalence of otomycosis among patients with a suspicion of infectious otitis in AmirAlam hospital as a tertiary referral medical center for ear, nose and throat disorders in Tehran, Iran. The prevalence of otomycosis was 28.39% (117 of 412). This finding is not in agreement with findings which reported the prevalence of otomycosis as 80%, 92%, 69%, and 78%, respectively (16, 19–21). However, some investigations reported the prevalence as 43%, 33.25%, 30.4%, and 19%, respectively, which are similar to our results (2, 22–24). These variations in results are probably due to different inclusion criteria. While, in some studies, only the individuals with a strong clinical suspicion of otomycosis are included, in our study all patients with a suspicion of infectious otitis were enrolled.

In this study, males were slightly more affected than females (64:53) in all age groups except the range of 66–75 yr. However, this difference was not statistically significant. Higher rate of male involvement is reported by some authors (20, 25), while, others reported female as the most affected gender (2, 23, 26). Moreover, the highest prevalence was recorded in the age group of 46–55 yr (30.77%) followed by 26–35 yr (23.07%). The highest prevalence of otomycosis is reported in 20–39 yr (23), 27–48 yr (22), 21–40 yr (20), 30–50 yr (26), and 31–40 yr (16) by some authors. These variations could be mainly due to the different patterns of age grouping or differences in geographical regions and climatic conditions.

Pruritus was the most common symptom (105, 89.74%) among 117 otomycosis patients. In agreement with our results, this complaint is reported as the predominant symptom in other studies (16, 19, 20, 25, 27). However, others reported ear fullness (28) and otalgia and otorrhea (15) as the main clinical symptom. Considering the divergent results in different studies and similarities between symptoms of bacterial and fungal otitis, the clinical symptoms are not specific for otomycosis. Therefore, clinical and laboratory findings should be used simultaneously for making the final decision on drug administration.

Auditory trauma/manipulation was the main predisposing factor observed in 83.76% of patients followed by a history of bacterial infection (33.33%), and swimming (30.77%). Similarly, injuries to the ear canal was reported as the most frequent predisposing factor of otomycosis (25, 27). This could be due to the defect in the skin caused by the injuries. Skin acts as the primary defense against various pathogens; therefore, lack of its normal structure facilitates the colonization and infection by different microorganisms including fungi.

During the laboratory analysis of specimens, a set of 133 fungal isolates including 92 (69.17%) molds and 41 (30.83%) yeasts were recovered from 117 patients. *A. niger* (n=50, 37.59%) was the most common isolated species followed by *A. flavus* (n=36, 27.06%). Among the yeast isolates which totally belonged to the genus *Candida*, *C. glabrata* (n= 25, 18.79%) was the most common species. In accordance with our findings, *A. niger* was reported as the most common species in some studies (20, 23, 25–27, 29). However, in other studies, *A. flavus* was reported as the leading fungal etiology of otomycosis (16, 19). In addition, *C. parapsilosis* was the most common species (24). Since in the most of studies, *A. niger* and *A. flavus* were specified as the most prevalent fungal etiology of otomycosis, uncommon species should not be ignored. In our study, *C. glabrata* was recovered from 25 (18.76%) patients. This is not a common finding in literature. Some of non-*albicans Candida* species are intrinsically resistant to azole antifungal drugs. Therefore, they could cause treatment failure and should be taken into account.

Mixed fungal otitis was identified in 16 patients in our study. Coinfection due to *A. niger* and *C. glabrata* was the most frequent pattern (7 cases), followed by *A. niger* and *A. flavus* (4 cases). In agreement with our results, two cases of mixed otomycosis caused by *Aspergillus* species and *C. albicans* were reported (28). Furthermore, five cases of mold and yeast coinfection were found among 88 otomycosis patients which were due to *A. flavus* and *C. albicans* (2 cases), *A. flavus* and *C. parapsilosis* (1 case), *A. flavus* and *C. guilliermondii* (1 case) and *A. flavus* and *C. famata* (1 case) (19). Regarding the endogenous nature of *Candida* species, the control and prevention of infections caused by them is completely different from those due to exogenous fungi such as *Aspergillus* species. This issue should be highlighted in treatment, control, and prevention of otomycosis. Therefore, treatment failure and recurrence of disease is not an unexpected result in the cases of otomycosis treated based on clinical symptoms and without the laboratory findings.

## Conclusion

Otomycosis was diagnosed in 117 of 412 patients with almost an equal distribution among male and female subjects. *A. niger* was the predominant species similar to the majority of other reports. High prevalence of *C. glabrata* and also 16 cases of mixed fungal otitis were identified. Regarding inter/intra species and genus differences in susceptibility patterns of fungi, it is preferred to perform laboratory analysis prior to empirical treatment of patients.

## Ethical considerations

Ethical issues (Including plagiarism, informed consent, misconduct, data fabrication and/or falsification, double publication and/or submission, redundancy, etc.) have been completely observed by the authors.
